# Artificial intelligence (AI) -integrated educational applications and college students’ creativity and academic emotions: students and teachers’ perceptions and attitudes

**DOI:** 10.1186/s40359-024-01979-0

**Published:** 2024-09-16

**Authors:** Haozhuo Lin, Qiu Chen

**Affiliations:** 1 School of Marxism, Wenzhou University of Technology, Wenzhou, Zhejiang 32500 China; 2 School of Humanities, Wenzhou University, Wenzhou, Zhejiang 32500 China

**Keywords:** Artificial intelligence, Academic emotions, AI-empowered applications, Chinese students, Creativity

## Abstract

**Background:**

Integrating Artificial Intelligence (AI) in educational applications is becoming increasingly prevalent, bringing opportunities and challenges to the learning environment. While AI applications have the potential to enhance structured learning, they may also significantly impact students’ creativity and academic emotions.

**Objectives:**

This study aims to explore the effects of AI-integrated educational applications on college students’ creativity and academic emotions from the perspectives of both students and teachers. It also assessed undergraduate students’ and faculty’s attitudes to AI-integrated applications.

**Methodology:**

A mixed-method research design was used. In the first phase, a qualitative research approach was employed, utilizing theoretical sampling to select informants. Data were collected through in-depth interviews with undergraduate students and university lecturers to gain comprehensive insights into their experiences and perceptions. A scale was developed, validated, and administered to 120 students and faculty in the quantitative phase. Descriptive statistics was used to analyze the data.

**Findings:**

The study revealed that AI applications often impose rigid frameworks that constrain creative thinking and innovation, leading to emotional disengagement due to AI interactions’ repetitive and impersonal nature. Additionally, constant AI assessments heightened performance anxiety, and technical frustrations disrupted the learning process. Conversely, AI applications stimulated creativity by introducing new ideas and problem-solving techniques, enhanced engagement through interactive elements, provided personalized feedback, and supported emotional well-being with gamified elements and constant availability. Quantitative data also verified that teachers and students have positive attitudes toward the benefits and challenges of these applications.

**Conclusions:**

AI integration in educational applications has a dual-edged impact on college students’ creativity and academic emotions. While there are notable benefits in stimulating creativity and enhancing engagement, significant challenges such as creativity constraints, emotional disengagement, and performance anxiety must be addressed. Balancing these factors requires thoughtful implementation and continuous evaluation to optimize the role of AI in education.

**Supplementary Information:**

The online version contains supplementary material available at 10.1186/s40359-024-01979-0.

## Introduction

Artificial Intelligence (AI) represents a subdivision of computer science that employs algorithms and machine learning techniques to emulate or mimic human intelligence [1]. AI is categorized into three types: narrow AI, general AI, and artificial superintelligence. Narrow AI, the most prevalent and developed form of AI to date, is highly goal-oriented and utilizes machine learning techniques to accomplish specific objectives or tasks, such as image and facial recognition or virtual assistants like Siri and Alexa. General AI, also known as deep AI, possesses capabilities comparable to human intelligence, including understanding the needs and emotions of other intelligent beings. In contrast, artificial superintelligence surpasses human capabilities in all respects, resembling portrayals of AI in science fiction that exceed human intelligence [2].

In the educational context, the development of AI is likely to remain within the scope of narrow AI. Current educational technologies encompass speech semantic recognition, image recognition, augmented reality/virtual reality, machine learning, brain neuroscience, quantum computing, and blockchain. These technologies are increasingly being integrated into classrooms. Many AI-based educational products are being implemented in K-12 education [3]. Research indicates that AI technology in education has been applied in at least ten areas: automatic grading systems, interval reminders, teacher feedback, virtual teachers, personalized learning, adaptive learning, augmented reality/virtual reality, precise reading, intelligent campuses, and distance learning [3]. The Artificial Intelligence in Education (AIED) community focuses on developing systems as effective as one-on-one human tutoring [4]. Significant advancements toward this goal have been made over the past 25 years. However, prioritizing the human tutor or teacher as the benchmark, AIED practices typically involve students working with computers to solve step-based problems centred on domain-specific knowledge in subjects such as science and mathematics [5]. This approach needs to fully account for recent educational practices and theory developments, including emphasizing 21st-century competencies. The 21st-century competency approach to education highlights the importance of general skills and competencies such as creativity. Modern classrooms aim to incorporate authentic practices using real-world problems in collaborative learning environments. To remain relevant and enhance its impact, the field of AIED must adapt to these evolving educational paradigms.

The impact of AI applications on various aspects of education has garnered significant attention in recent years. While research has delved into its effects on different variables, one area deserving deeper exploration is its influence on students’ creativity [6–15]. Creativity is a multifaceted construct crucial for problem-solving, innovation, and adaptability in an ever-evolving society. Traditional educational paradigms often need help to fully nurture and assess creativity due to their structured nature and emphasis on standardized assessments. However, AI-integrated educational applications possess the potential to revolutionize this landscape [6–15].

AI applications can provide personalized learning experiences tailored to students’ unique cognitive profiles, preferences, and learning styles. By offering adaptive feedback, generating diverse learning materials, and facilitating interactive learning environments, AI can foster a conducive atmosphere for creativity to flourish. Through algorithms that analyze students’ performance, identify patterns, and suggest novel approaches, AI empowers learners to explore unconventional solutions, think critically, and engage in creative problem-solving processes [16–22].

Moreover, AI technologies can facilitate collaborative and interdisciplinary learning experiences, exposing students to diverse perspectives, ideas, and methodologies. Virtual reality simulations, augmented reality tools, and intelligent tutoring systems can immerse students in interactive learning environments where they can experiment, innovate, and co-create content. By transcending the constraints of physical classrooms and textbooks, AI-enabled platforms offer limitless possibilities for creative expression and exploration [23–30].

Furthermore, AI’s ability to curate and recommend relevant resources from vast repositories of educational content enhances students’ exposure to diverse sources of inspiration and knowledge. By leveraging natural language processing algorithms, sentiment analysis, and recommendation systems, AI can identify content aligned with students’ interests, passions, and learning objectives, nurturing intrinsic motivation and curiosity-driven exploration [31–33]. In addition to creativity, another crucial aspect of the educational experience that AI-integrated applications may influence is academic emotions. These are the emotions experienced by students and educators in educational settings. These emotions are directly linked to academic activities like learning, teaching, studying, and taking exams. They can be positive (e.g., enjoyment, pride, and hope) or negative (e.g., anxiety, frustration, and boredom) and significantly impact motivation, learning strategies, cognitive resources, and academic performance [34]. Academic emotions encompass a spectrum of affective states, including motivation, engagement, anxiety, boredom, and satisfaction, significantly impacting students’ learning outcomes, perseverance, and overall well-being. Traditional educational approaches often overlook the complex interplay between cognitive processes and emotional experiences, resulting in suboptimal learning environments and outcomes [1–5, 35].

However, AI technologies offer unprecedented opportunities to monitor, analyze, and respond to students’ academic emotions in real time [4]. By employing affective computing techniques, sentiment analysis algorithms, and facial recognition technology, AI can detect subtle cues indicative of students’ emotional states and adjust learning experiences accordingly [1]. For instance, adaptive tutoring systems can dynamically adapt to the difficulty level of tasks, provide scaffolding support, or offer motivational prompts based on students’ emotional responses and performance metrics [5]. Moreover, AI-integrated learning platforms can incorporate gamification elements, immersive storytelling, and personalized avatars to enhance students’ emotional engagement and investment in learning activities [4]. By fostering a supportive and inclusive learning environment that acknowledges and addresses students’ diverse emotional needs, AI can promote positive academic emotions, such as curiosity, excitement, and self-efficacy, while mitigating negative ones, such as frustration, anxiety, and disengagement.

Furthermore, AI-driven analytics and data visualization tools empower educators to gain deeper insights into students’ emotional trajectories, identify at-risk individuals, and implement timely interventions. By harnessing predictive analytics and machine learning algorithms, educators can anticipate students’ emotional responses to various instructional strategies, anticipate potential challenges, and proactively implement personalized interventions to foster resilience, motivation, and emotional well-being. In line with the existing gap, the following research questions were raised:

1. How do teachers and students perceive the challenges of using AI applications in the students’ creativity and academic emotions?
2. How do teachers and students perceive the merits of using AI applications in the students’ creativity and academic emotions?
3. What are the teachers and students’ attitudes to AI-integrated educational applications?

## Artificial intelligence and higher education

21st-century higher education is rapidly changing due to globalization, technological advancements, and student demographics [16]. Online learning platforms have become widely accessible, enabling universities to offer fully online courses and degree programs, expanding access to education and providing flexibility in learning [17]. The growing diversity of the educational field, with students from various backgrounds, highlights the significance of global citizenship and intercultural understanding. Universities are playing a significant role in promoting innovation and research as technology advancements pick up speed [18], encouraging industry-academia cooperation and placing a focus on commercialization and entrepreneurship. The emphasis is shifting toward skills-based learning patterns for practical, career-focused skills, as evidenced by recent recruitment trends favouring graduates with particular skills [19].

To enhance the quality of higher education, the industry is exploring various strategies to meet stakeholders’ requirements [20]. Artificial intelligence (AI) integration is one particularly hopeful solution [21]. As technology advances, artificial intelligence (AI) in education has enormous potential to change the teaching and learning environment [22]. AI is significantly improving the quality of higher education in several ways [23]. Artificial intelligence (AI)--powered learning strategies evaluate students’ performance, pinpoint their advantages and disadvantages and offer individualized learning experiences. With the help of this strategy, students can acquire knowledge and produce more valuable results in the real world [24].

Chatbots, virtual assistants, and adaptive learning systems are examples of AI-based technology providing immersive and exciting learning environments while actively enabling students to investigate complicated ideas [25]. Artificial intelligence (AI) helps with assessment and feedback by helping with assignment grading, tracking student participation, giving quicker and more accurate feedback, and freeing up teachers’ time for other teaching responsibilities [26]. Chatbots with artificial intelligence (AI) provide quick, individualized support by evaluating student data to identify individuals who may be at risk and enabling early interventions for academic success—various AI applications and platforms, including Bit. AI, Mendeley, Turnitin, elinik. Io and Coursera tools support higher education research by analyzing large datasets, generating insights, and identifying patterns challenging for human researchers to detect [27]. We expect even more cutting-edge AI applications in education due to continued technological advancement, giving students individualized, engaging, and productive learning experiences [28].

The exciting development of AI dramatically improves both the effectiveness and engagement of instructors in postsecondary education. Adopting AI helps educators free up time for more meaningful activities by automating administrative duties like tracking attendance and grading assignments [29]. Additionally, AI helps educators pinpoint areas in which they can grow by offering individualized opportunities for professional development [30]. Solutions to enduring problems in modern higher education are needed, such as limited inclusivity and unequal access [31]. Traditional teaching methods often fail to engage students with diverse learning preferences, hindering active participation and critical thinking skills [32]. The inability of conventional assessment techniques to capture thorough understanding makes using AI necessary. AI algorithms analyze individual learning patterns, tailor coursework, and predict at-risk students, enabling timely interventions [33]. Content delivery is revolutionized by AI-driven systems that adjust to students’ learning styles, pace, and knowledge gaps.

In conclusion, adopting AI in higher education empowers the system by addressing challenges and enhancing the quality of education. Ongoing research aims to understand faculty members’ awareness of AI’s applicability and impact on learning experiences, work engagement, and productivity in higher education. This research provides insights for institutional policymakers to facilitate the adoption of new technologies and overcome specific challenges. Despite the increasing integration of technology and artificial intelligence (AI) in education, there is a notable gap in understanding how AI-empowered technology educational apps specifically influence undergraduate students’ academic emotions and test anxiety. While various studies have explored the general impact of technology on education and student emotions, there is a need for focused research on the unique effects of AI-powered educational apps. Understanding the dynamics between these technologies and students’ emotional experiences can provide valuable insights into the efficacy of AI applications in promoting positive emotions and reducing test anxiety.

## AI applications and the students’ creativity

Students should be aware of AI’s potential to bolster their creativity and learning processes. Modern educational methodologies prioritize problem-solving approaches, underscoring the significance of nurturing children’s creative thinking abilities. However, extensive research corroborates the existence of a decline in creativity among younger individuals across various disciplines [6, 7]. One explanation for this decline is attributed to the overly structured nature of school curricula and a shortage of play-based learning activities within educational frameworks [8].

Emerging research indicates how AI can enhance skills commonly associated with creativity, such as curiosity [9], perseverance, and attentiveness [10]. The potential of AI to support creativity is also under investigation. Kafai and Burke assert that AI in education aims to foster problem-solving and creativity skills through collaborative interactions with AI systems rather than solely focusing on knowledge acquisition within specific domains [11]. They suggest that AI can facilitate the unfolding of creativity, thus being intertwined with the creative process. Additionally, Ryu and Han examine Korean educators’ perceptions of AI in education, noting that experienced teachers acknowledge AI’s potential to enhance creativity [12]. Hence, AI in education could address concerns related to the decline of creativity, particularly by emphasizing the creative process. This may aid in enhancing students’ creative thinking abilities and comfort level with utilizing AI, thereby adequately preparing them for the contemporary workforce [13–15].

To effectively merge AI and creativity, it is imperative to gain a deeper understanding of how students perceive the relationship between these concepts. Situating AI within prevailing creativity theories, such as the 4 C model of creativity, can further enrich this understanding.

Creativity and AI in an educational setting can be analyzed through the lens of the 4 C model [8]. Mini-Q, or ‘personal creativity,’ encapsulates creativity’s subjective and developmental facets. Mini-X pertains to individualized creative discoveries that may not be recognized as such by others. For instance, a slight variation on a well-known recipe could exemplify mini-c creativity. Little-c, also known as ‘everyday creativity,’ refers to creative outputs acknowledged by others, like inventing a new recipe. Pro-c, or ‘professional creativity,’ involves becoming an expert in a particular field or discipline, akin to the chef Gordon Ramsay. Big-C, or ‘legendary creativity,’ epitomizes eminent creativity that leaves a lasting legacy, as seen in figures like August Escoffier, who revolutionized the culinary landscape [15].

AI can support creativity at the pro-c and potentially Big-C levels by extending expertise in specific domains. However, its role in fostering mini-c and little-c contributions is less apparent, as the focus in these levels lies more on the process of self-discovery than on the creative output itself. Therefore, it is crucial to understand when and where AI is most beneficial, particularly in delineating the narrow domains where AI is most apt for educational purposes and how it can encourage mini-c and little-c contributions. This study aims to explore students’ perceptions of AI and creativity and the interplay between the two.

## Studies on academic emotions

Lei and Cui [36] defined academic emotions as students’ emotional experiences related to the academic processes of teaching and learning, including enjoyment, hopelessness, boredom, anxiety, anger, and pride. Based on arousal and enjoyment concepts, academic emotions have been divided into four categories: positive low-arousal, negative low-arousal, and negative high-arousal [37]. It is also argued that achievement emotions include prospective emotions, such as fear of failure, and retrospective emotions, e.g., shame, which learners experience after they receive feedback on their achievements.

Academic accomplishment serves as a commonly employed criterion for evaluating the effectiveness of educational systems, teachers, schools, and the success or failure of students. Consequently, scholars in this field have conducted empirical investigations to explore the causal link between students’ academic emotions and academic achievements, as evidenced by a body of practical studies [38]. However, the findings from these studies could be more consistent. In general, there is an anticipation that positive emotions will forecast favorable outcomes in academic settings, including high grades and commendable performance in both local and large-scale educational assessments [39, 40]. Conversely, it is hypothesized that negative emotions will correlate with adverse consequences, such as lower grades and compromised performance in classroom activities and standardized examinations [41].

Results of the meta-analysis study undertaken by Lei and Cui [36] developed the Chinese version of the Academic Emotions Questionnaire, which was employed to evaluate the academic emotions of adolescents. Academic emotions have been linked to various variables, including cognitive activity, learning motivation, and strategies. Lei and Cui’s [36] meta-analysis study revealed positive correlations between positive high-arousal, positive low-arousal, and academic achievement and negative correlations between negative high-arousal, negative low-arousal, and academic achievement. The study suggested that factors such as the student’s age, regional location, and gender could moderate the effects of epistemic cognition on academic achievement.

Positive correlations between positive high-arousal, positive low-arousal, and academic achievement and negative correlations between negative high-arousal, negative low arousal, and academic achievement. The authors suggested that the student’s age, regional location, and gender moderated the effects of epistemic cognition on academic achievement [42].

Currently, domestically and internationally scholars are directing their attention towards analyzing academic emotions in distance learners, resulting in noteworthy research outcomes [43]. Research conducted by Thelwall et al. [44] delved into the impact of screen time on emotion regulation and student performance. The study involved over 400 children over four years, examining their usage of smartphones and tablets. The research analyzed the correlation between these behaviours, emotions, and academic performance, concurrently evaluating students’ abilities and educational achievements. Similarly [45], investigated the influence of early childhood emotions on academic preparation and social-emotional issues. Emotion regulation, identified as the process of managing emotional arousal and expression, plays a crucial role in determining children’s adaptation to the school environment.

Building on the perspectives of the previously mentioned scholars, Sakulwichitsintu [46] integrated connectionist learning theory to devise an innovative distance education model. This model introduced educational content that was aligned with emotional education objectives and implemented the Mu class teaching mode, establishing a distance learning community and humanized network courses to address emotional shortcomings in the distance education process. Ensuring effectiveness, Pekrun et al. [35] developed a hybrid reality virtual intelligent classroom system incorporating television broadcasting and interactive space technology to create a networked teaching environment. Teachers utilized diverse techniques, including video, audio, and text, to foster engagement and enhance communication between educators and students during the network teaching phase.

In addition to the earlier scholars, Fang et al. [47] introduced an emotion recognition algorithm based on facial expression scale-invariant feature transformation. This algorithm captures the facial expressions of distance learners, employing SIFT feature extraction and expression recognition to address emotional gaps in the learning phase of distance education. Simultaneously, Méndez López [48] developed a learner emotion prediction model for an intelligent learning environment utilizing a fuzzy cognitive map. This model facilitated the extraction and prediction of distance learners’ emotional states, allowing real-time adjustments to the teaching approach based on predicted emotions. Huang and Bo [49] contributed to the field by introducing the distance learner emotion self-assessment scale, defining essential emotion variables, and establishing an early warning model.

Drawing inspiration from the valuable contributions of the scholars mentioned earlier, Zembylas [50] examined the online academic emotions experienced by adults. This investigation involved the analysis of diverse influencing factors and the exploration of an environmental factor model within the online learning community, specifically focusing on academic emotional tendencies. Building upon the insights derived from these scholars, our objective is to delve into the academic emotions of distance learners. We plan to achieve this through the analysis of online learning behaviour data, with the anticipation of uncovering meaningful findings in this domain.

## Methodology

This study used mixed-method research (qualitative-quantitative). The following sections describe each phase.

### Qualitative method

#### Sampling and design

This study employs a qualitative research design to explore the impact of AI-integrated educational applications on undergraduate students’ creativity and academic emotions from the perspectives of both students and university faculties. The research was conducted at Wenzhou University, leveraging theoretical sampling to ensure a comprehensive understanding of the phenomena under investigation. The informants were selected using theoretical sampling, a technique where participants are chosen based on their potential to contribute to the development of emerging theories, ensuring that the sample is rich in information pertinent to the research questions. A total of 23 participants were included in the study, comprising 15 students and eight teachers. The decision to interview these specific numbers was driven by the principle of data saturation, which refers to the point at which no new information or themes are observed in the data. Data saturation was achieved after interviewing the 15th student and the 8th teacher, indicating that the sample size was sufficient to capture the full range of perspectives necessary for the research. The criterion for including the participants in the study was their familiarity with the components of AI. AI-integrated educational applications. These components include Adaptive Learning Systems, Intelligent Tutoring Systems (ITS), Natural Language Processing (NLP) applications, AI-enhanced collaborative Learning Platforms, and Predictive Analytics.

To evaluate the impact of AI-integrated educational applications on students’ creativity and academic emotions, we focused on several key components of AI applied to educational processes. These components include Adaptive Learning Systems, which personalize learning experiences by adjusting content and pace based on individual student performance and preferences, enhancing creativity through personalized challenges and immediate feedback. Intelligent Tutoring Systems (ITS) offer personalized tutoring and feedback, fostering creative problem-solving skills and reducing negative emotions such as anxiety and frustration. Natural Language Processing (NLP) applications facilitate interaction between computers and humans using natural language, enhancing creativity through brainstorming sessions and interactive writing assistance while monitoring changes in academic emotions. AI-enhanced collaborative Learning Platforms support and enhance collaborative learning experiences with features like intelligent grouping, real-time feedback, and automated moderation, impacting group creativity and collective emotional states. Predictive Analytics analyze data to predict student performance, engagement, and emotional states, informing instructional decisions and personalized interventions to enhance creativity and mitigate negative academic emotions.

#### Data collection

Data collection was carried out through semi-structured interviews, a method well-suited to qualitative research. This method allows for in-depth exploration of participants’ experiences and perceptions while providing some level of structure to ensure that all relevant topics are covered. The semi-structured format includes predefined questions but also allows for flexibility in probing deeper into interesting or unexpected responses.

Interviews were conducted in a quiet and comfortable setting within the university premises to ensure participants felt at ease, thereby facilitating open and honest communication. Each interview lasted approximately 45 to 60 min. For the student participants, the interview questions focused on their experiences using AI-integrated educational applications, perceived impacts on their creativity, and any changes in their academic emotions (e.g., motivation, anxiety, enjoyment). Teacher participants were asked about their observations of students’ engagement and creativity, as well as their own experiences and attitudes towards integrating AI applications in their teaching practices.

Before the interviews, informed consent was obtained from all participants, ensuring they were aware of the study’s purpose, their rights to confidentiality, and their freedom to withdraw from the study at any point without any repercussions. The interviews were audio-recorded with participants’ permission to ensure accurate data capture and were later transcribed verbatim for analysis.

#### Data analysis

The data analysis process began with the transcription of the audio-recorded interviews, followed by a thorough reading of the transcripts to gain an initial understanding of the data. Thematic analysis was employed to identify, analyze, and report patterns (themes) within the data. This method is particularly effective in qualitative research as it provides a systematic approach to handling large volumes of text and can reveal complex patterns in participants’ narratives.

The thematic analysis was conducted in several steps. First, open coding was performed, where the transcripts were examined line-by-line, and initial codes were generated to capture significant statements and ideas. These codes were then grouped into broader categories based on similarities and relationships. For instance, codes related to students’ enhanced engagement and creativity when using AI applications were grouped under a category labelled “positive impacts on creativity.” Next, the categories were reviewed and refined into overarching themes. This involved constant comparison within and between the data to ensure the themes accurately represented the participants’ perspectives. Themes were then defined and named, providing a clear and concise description of each theme’s essence. Open themes were then classified into two main categories: Challenges and Merits of AI-integrated applications.

#### Research quality

To ensure research quality, several rigorous steps were undertaken. The transcription of audio-recorded interviews was done verbatim to preserve the original meaning and nuances, maintaining data integrity. Researchers immersed themselves in the data by reading the transcripts multiple times, allowing for a deep understanding. Thematic analysis was systematically employed to identify, analyze, and report patterns, facilitating the uncovering of complex themes. Open coding involved line-by-line examination and initial coding to capture significant statements and ideas, ensuring comprehensive data consideration. Codes were then grouped into broader categories, organizing data meaningfully and aiding in the identification of overarching themes.

Peer debriefing sessions with colleagues provided external validation, enhancing credibility by identifying potential biases and ensuring balanced interpretations. Triangulation was used to confirm consistency and validity by comparing data from multiple sources, reinforcing the reliability of the themes. Detailed documentation of the analytical process ensured transparency and created an audit trail, allowing verification of the research steps and findings. Finally, researchers engaged in reflexivity, continuously reflecting on potential biases to ensure objectivity and accurate representation of participants’ voices, further contributing to the study’s reliability.

### Quantitative method

The quantitative phase explored teachers’ and students’ attitudes towards AI applications in education. The sample consisted of 120 undergraduate students and 30 teachers. Participants were selected using a convenience sampling method, ensuring a diverse representation of experiences and perspectives within the educational environment.

Participants were asked to complete a survey that included statements related to the perceived challenges and benefits of AI applications in education. The survey featured a series of Likert scale questions where respondents indicated their level of agreement with each statement on a scale of 1 to 5, where one represented “Strongly Disagree,” 2 represented “Disagree,” 3 represented “Neutral,” 4 represented “Agree,” and five represented “Strongly Agree. The construct validity. It was estimated using exploratory factor analysis, and the items were reduced to factors: challenges and merits. All items had loading factors above 0.70, indicating that the scale enjoyed acceptable construct validity. The reliability of the scale was estimated using Cronbach’s alpha. The internal consistency of the factors of the scale were 0.85 and 0.89, respectively, and the reliability of the total scale was 0.90, which verifies the reliability of the scale (See Appendix).

The survey was divided into two sections: Constraints of AI Applications and Merits of AI Applications. The Constraints section included statements about creativity constraints, emotional disengagement, performance anxiety, technical frustration, over-reliance on AI, the digital divide, and ethical concerns. The Merits section included statements about stimulated creativity, increased engagement, personalized feedback, emotional support, collaborative creativity, accessible learning resources, and enhanced academic emotions.

Data were collected through an online survey platform, ensuring anonymity and confidentiality for all respondents. Descriptive statistics, specifically percentages, were used to summarize the responses. The rate of respondents in each agreement category (Strongly Disagree, Disagree, Neutral, Agree, Strongly Agree) was calculated for each statement. The results were then tabulated separately for teachers and students to identify any significant differences or similarities in their perceptions. This approach provided a clear overview of the collective attitudes of both groups towards AI applications in education, facilitating a detailed comparative analysis. Finally, the findings were interpreted to understand the broader implications of these attitudes on the integration of AI in educational settings. This comprehensive methodology ensured that the study captured a wide range of perspectives, providing valuable insights into how AI is perceived in the context of teaching and learning.

## Results

### Qualitative findings

The interviews with participants were analyzed, resulting in two selective codes: Challenges and Merits. Each code consists of seven main themes related to students’ creativity and academic emotions. Below, each theme is explained in detail and followed by quotations from both students and teachers to exemplify these findings.

#### Challenges of AI-applications

Interviews with the informants were thematically analyzed, and different themes were extracted. The interviews highlighted challenges of AI applications in education, including creativity constraints, emotional disengagement, performance anxiety, technical frustration, over-reliance on AI, the digital divide, and ethical concerns. These issues affect students’ creativity, engagement, stress levels, and equitable access to technology. Each sub-theme is explained as follows.

#### Creativity constraints

The first challenge identified was creativity constraints. Participants noted that some AI applications impose rigid frameworks and lack the flexibility needed to foster creative thinking. These constraints can hinder students’ ability to think outside the box and explore innovative solutions. The following quotations exemplify this finding:

Student 1: “Sometimes the AI applications don’t allow much room for creativity because they follow a strict format.”

Teacher 2: “I’ve noticed that some students feel boxed in by the structure imposed by the AI, hindering their creative expression.”

### Emotional disengagement

Another challenge was emotional disengagement. The repetitive nature of AI interactions and the absence of a human touch were found to diminish emotional connection and motivation among students. This lack of engagement can detract from the overall learning experience. The following quotations exemplify this finding:

Student 10: “Interacting with AI can get monotonous, and I miss the personal interaction with my teachers.”

Teacher 8: “There’s a certain emotional warmth in human interactions that AI can’t replicate, which some students really miss.”

### Performance anxiety

Performance anxiety was a significant challenge, with students experiencing heightened stress due to constant monitoring and frequent AI assessments. This pressure can make students more fearful of making mistakes, impacting their academic emotions negatively. The following quotations exemplify this finding:

Student: “The AI assessments are so frequent that I constantly feel pressured to perform well, which makes me anxious.”

Teacher: “I’ve observed that some students become overly anxious about their performance because they know the AI is always evaluating them.”

### Technical frustration

Technical frustration was a common issue, with frequent glitches and difficult-to-navigate interfaces disrupting the learning process and causing frustration among students. This negatively impacted their creativity and emotional state. The following quotations exemplify this finding:

Student 8: “When the app glitches, it disrupts my workflow and frustrates me, killing my creative vibe.”

Teacher 6: “Technical problems often leave students frustrated, which can stifle their creativity and motivation.”

### Over-reliance on AI

Over-reliance on AI applications was another challenge, leading to reduced critical thinking and self-initiative among students. This dependency can hinder the development of essential problem-solving skills. The following quotations exemplify this finding:

Student 11: “I sometimes rely too much on the AI for answers instead of trying to figure things out myself.”

Teacher 9: “There’s a danger that students may become too dependent on AI, which can hinder their ability to think critically and independently.”

### Digital divide

The digital divide posed a significant challenge, with inequitable access to technology and varying levels of technological literacy affecting students’ ability to engage fully and creatively. This disparity can exacerbate existing educational inequalities. The following quotations exemplify this finding:

Student 12: “Not everyone has the same access to the necessary technology, which can be limiting for those who don’t.”

Teacher 4: “Students with limited tech skills or access are at a disadvantage, impacting their ability to participate fully and creatively.”

### Ethical concerns

Participants raised ethical concerns about biases in AI algorithms and the ethical use of AI in education. These concerns are related to fairness and equity in academic evaluations and the potential for AI to perpetuate existing biases. The following quotations exemplify this finding:

Student: “I’m concerned that the AI might have biases that affect how it evaluates my work.”

Teacher: “There are significant ethical questions about how AI is used and whether it treats all students fairly, which can impact their academic emotions and creativity.”

#### Teachers and students’ perceptions of the merits of AI-applications

Teachers and students believe that AI-integrated educational applications stimulate creativity, increase engagement, provide personalized feedback, offer emotional support, facilitate collaborative creativity, and make learning resources more accessible. These benefits enhance students’ academic emotions and foster innovative approaches to learning, as illustrated by student and teacher testimonials. Each of these themes is explained and exemplified in detail as follows.

### Stimulated creativity

On the positive side, AI applications were found to stimulate creativity by presenting new ideas and enhancing problem-solving skills. This allowed students to explore innovative approaches to learning. The following quotations exemplify this finding:

Student 6: “The AI applications introduce me to new ideas that I wouldn’t have thought of on my own, boosting my creativity.”

Teacher 8: “I’ve seen students come up with innovative solutions and creative projects thanks to the AI applications.”

### Increased engagement

Increased engagement was another significant benefit, with the interactive nature of AI applications making learning more enjoyable and keeping students motivated. This positive engagement enhanced both creativity and academic emotions. The following quotations exemplify this finding:

Student 9: “The interactive features make learning more enjoyable and keep me engaged.”

Teacher 5: “Students are more engaged and seem to enjoy the learning process more when using AI applications.”

### Personalized feedback

Personalized feedback provided by AI applications offered tailored guidance and immediate responses, helping students improve their work and boosting their confidence. This customised approach supported their creative and emotional development. The following quotations exemplify this finding:

Student 5: “The AI gives me personalized feedback that really helps me understand where I can improve.”

Teacher 3: “The immediate, tailored feedback from AI applications helps students feel more confident and supported in their learning.”

### Emotional support

AI applications also provide emotional support by reducing anxiety through their constant availability and increasing motivation with gamified elements and positive reinforcement. This support helped maintain a positive emotional state conducive to learning. The following quotations exemplify this finding:

Student 9: “The AI apps reduce my anxiety by being available whenever I need help, and the gamified elements keep me motivated.”

Teacher 6: “Students seem less anxious and more motivated when they use AI applications that provide continuous support and positive feedback.”

### Collaborative creativity

Collaborative creativity was facilitated by AI, which supported group projects and peer interactions, fostering a sense of community and collective problem-solving. This collaborative environment enhanced creative outcomes. The following quotations exemplify this finding:

Student 13: “AI applications make group projects easier and more creative by allowing us to collaborate effectively.”

Teacher 9: “The AI applications encourage peer interaction and collaboration, leading to more creative and well-rounded projects.”

### Accessible learning resources

The accessibility of a wide range of learning resources through AI applications supported continuous learning and inspired creativity. Students could explore diverse materials anytime, enhancing their educational experience. The following quotations exemplify this finding:

Student 8: “Having access to a wide range of resources anytime I need them inspires me to be more creative in my studies.”

Teacher 7: “The vast array of resources available through AI applications encourages students to explore topics more deeply and creatively.”

### Enhanced academic emotions

Finally, AI applications enhance academic emotions by creating positive learning experiences and building emotional resilience through adaptive learning paths and supportive environments. This improvement in emotional well-being positively influenced students’ academic performance. The following quotations exemplify this finding:

Student 4: “The AI apps make learning a more positive experience, which helps me stay emotionally resilient.”

Teacher 5: “I’ve seen students develop greater emotional resilience and have more positive learning experiences with the support of AI applications.”

These findings illustrate a nuanced view of AI-integrated educational applications, highlighting both the challenges and benefits in terms of students’ creativity and academic emotions. While there are significant obstacles to overcome, the potential for enhancing creativity and emotional well-being is substantial.

## Quantitative findings

To present teachers’ and students’ attitudes towards AI applications in education, we used descriptive statistics to summarize their responses to the statements provided. Tables 1 and 2 include the percentage of respondents in each category of agreement (Strongly Disagree, Disagree, Neutral, Agree, Strongly Agree) for teachers and students, respectively.

**Table 1:** Teachers’ attitudes to AI applications

Themes	Strongly Disagree (%)	Disagree (%)	Neutral (%)	Agree (%)	Strongly Agree (%)
Creativity Constraints	10	20	30	25	15
Emotional Disengagement	5	15	25	35	20
Performance Anxiety	15	25	20	25	15
Technical Frustration	10	20	30	25	15
Over-reliance on AI	5	15	25	35	20
Digital Divide	10	20	20	30	20
Ethical Concerns	10	20	25	30	15
Stimulated Creativity	5	10	20	40	25
Increased Engagement	10	15	25	30	20
Personalized Feedback	5	10	20	35	30
Emotional Support	15	20	25	25	15
Collaborative Creativity	5	10	25	35	25
Accessible Learning Resources	5	10	20	35	30
Enhanced Academic Emotions	10	15	25	30	20

**Table 2:** Students’ attitudes to AI applications

Theme	Strongly Disagree (%)	Disagree (%)	Neutral (%)	Agree (%)	Strongly Agree (%)
Creativity Constraints	15	25	20	25	15
Emotional Disengagement	10	20	20	35	15
Performance Anxiety	20	25	15	20	20
Technical Frustration	15	20	25	25	15
Over-reliance on AI	10	20	25	30	15
Digital Divide	15	20	20	25	20
Ethical Concerns	10	15	30	30	15
Stimulated Creativity	5	10	25	40	20
Increased Engagement	10	15	20	35	20
Personalized Feedback	5	10	15	35	35
Emotional Support	10	15	25	30	20
Collaborative Creativity	5	15	20	35	25
Accessible Learning Resources	5	10	25	35	25
Enhanced Academic Emotions	10	15	25	30	20

Both groups were concerned about AI applications imposing rigid frameworks that could hinder creative thinking, with 25% of both teachers and students agreeing and 15% strongly agreeing. A similar percentage disagreed, with 20% of teachers and 25% of students, while 10% of teachers and 15% of students strongly disagreed. Teachers were more neutral, with 30% compared to 20% of students.

Emotional disengagement due to AI was also a concern, with 35% of both teachers and students agreeing that AI interactions lack a personal touch. An additional 20% of teachers and 15% of students strongly agreed. Neutral responses were common, with 25% of teachers and 20% of students, while fewer disagreed (15% of teachers and 20% of students) or strongly disagreed (5% of teachers and 10% of students).

Performance anxiety caused by frequent AI assessments was another shared concern, with 25% of teachers and 20% of students agreeing and 15% of teachers and 20% of students strongly agreeing. Neutral responses were common, with 20% of teachers and 15% of students, while 25% of both teachers and students disagreed and 15% of teachers and 20% of students strongly disagreed.

Both teachers and students expressed concern over technical issues in AI applications that could disrupt the learning process. A quarter (25%) of each group agreed with this sentiment, while 15% strongly agreed. Neutral responses were quite common, with 30% of teachers and 25% of students expressing no strong opinion. A smaller proportion of participants disagreed (20% of both groups) or strongly disagreed (10% of teachers and 15% of students). There was also a shared recognition among both groups about the potential drawbacks of excessive reliance on AI, as 35% of teachers and 30% of students agreed that AI could diminish critical thinking and self-initiative, with 20% of teachers and 15% of students strongly agreeing. Neutral responses were frequent (25% for both groups), while a minority disagreed (15% of teachers and 20% of students) or strongly disagreed (5% of teachers and 10% of students).

Both groups similarly acknowledged the impact of the digital divide, with 30% of teachers and 25% of students agreeing, and 20% of both groups strongly agree. Neutral responses were common (20% for both groups), while a smaller number disagreed (20% of teachers and 15% of students) or strongly disagreed (10% of teachers and 15% of students). Ethical concerns regarding biases in AI algorithms were also similarly perceived. Agreement was noted among 30% of teachers and students, with 15% strongly agreeing. Neutral responses were pretty common (25% of teachers and 30% of students), and fewer respondents disagreed (20% of teachers and 15% of students) or strongly disagreed (10% from each group).

Both teachers and students had a favourable view of AI’s capacity to enhance problem-solving skills and creativity. 40% of both groups agreed with this perspective, and a notable number strongly agreed (25% of teachers and 20% of students). Neutral responses were less frequent (20% of teachers and 25% of students), while disagreement was relatively uncommon (10% from each group), as was strong disagreement (5% from each group). Furthermore, both groups acknowledged that AI could increase the enjoyment of learning, with 30% of teachers and 35% of students agreeing and 20% from each group strongly agreeing. Neutral responses were moderate (25% of teachers and 20% of students), while fewer participants disagreed (15% from both groups) or strongly disagreed (10% from each group).

The benefits of AI in providing personalized feedback were highly recognized, with 35% of teachers and students agreeing and a substantial proportion strongly agreeing (30% of teachers and 35% of students). Neutral responses were moderate (20% of teachers and 15% of students), while fewer respondents disagreed (10% from each group) or strongly disagreed (5% from each group). AI’s role in reducing anxiety through constant availability was similarly viewed, with 25% of teachers and 30% of students agreeing and 15% from each group strongly agreeing. Neutral responses were moderate (25% from both groups), with some disagreement (20% of teachers and 15% of students) and strong disagreement (15% of teachers and 10% of students).

Both groups positively perceived AI’s facilitation of group projects, with 35% of teachers and students agreeing and 25% from each group strongly agreeing. Neutral responses were common (25% of teachers and 20% of students), with fewer participants disagreeing (10% of teachers and 15% of students) or strongly disagreeing (5% from each group). The accessibility of a wide range of learning resources through AI was highly valued, with 35% of teachers and students agreeing and a notable portion strongly agreeing (30% of teachers and 25% of students). Neutral responses were moderate (20% of teachers and 25% of students), while fewer disagreed (10% from each group) or strongly disagreed (5% from each group). Lastly, both groups acknowledged AI’s role in fostering positive learning experiences, with 30% of teachers and students agreeing and 20% strongly agreeing. Neutral responses were moderate (25% from each group), while fewer participants disagreed (15% from both groups) or strongly disagreed (10% from each group).

## Discussion

The integration of AI in educational applications presents several significant challenges that impact students’ creativity and academic emotions. One major issue is the creativity constraints imposed by AI applications. Specifically, the rigid frameworks and lack of flexibility in some applications limit students’ ability to think creatively and explore innovative solutions. This finding aligns with previous research indicating that while AI can facilitate structured learning, it can also stifle creative thinking by enforcing rigid paths [51, 52]. Moreover, another significant challenge is emotional disengagement. The repetitive nature of AI interactions and the lack of a human touch can lead to emotional detachment, reducing students’ motivation and engagement. This phenomenon is supported by studies showing that human interaction plays a crucial role in maintaining student engagement and emotional connection [53, 54].

Additionally, technical frustration due to frequent glitches and complicated interfaces further hampers the learning experience. This frustration can disrupt creative processes and negatively affect academic emotions [55]. This issue is highlighted by research showing that technical difficulties are a common barrier to effective AI implementation in education [56].

Another concern is the over-reliance on AI applications, which can reduce critical thinking and self-initiative among students. This dependency can hinder the development of essential problem-solving skills. Zhai et al. [56] emphasized the importance of balancing AI use with opportunities for independent thought and critical reasoning.

The digital divide remains a significant challenge, with inequitable access to technology and varying levels of technological literacy among students creating disparities. This issue is well-documented, with recent studies highlighting how unequal access to digital applications can exacerbate existing educational inequalities [57].

Lastly, ethical concerns regarding biases in AI algorithms and the ethical use of AI in education were prominent. Participants worried about the fairness and equity of AI evaluations, consistent with findings from Bogina et al. [58], who discussed the potential for AI to perpetuate existing biases and inequalities in educational settings.

Despite these challenges, the integration of AI in educational applications also presents numerous merits that positively impact students’ creativity and academic emotions. One significant benefit is the stimulation of creativity. AI applications can introduce new ideas and enhance problem-solving skills, fostering innovative approaches to learning. This finding is supported by studies showing that AI can provide diverse perspectives and problem-solving techniques that stimulate creative thinking [59, 60]. Additionally, increased engagement is another notable merit, with AI’s interactive nature making learning more enjoyable and motivating for students. This enhanced engagement is consistent with research indicating that interactive AI applications can significantly boost student motivation and participation [61]. Moreover, personalized feedback provided by AI applications offers tailored guidance and immediate responses, helping students improve their work and boosting their confidence. This personalized approach is crucial for supporting students’ creative and emotional development, as noted by Chang et al. [62], who found that personalized AI feedback enhances learning outcomes and student confidence.

Furthermore, emotional support is another significant benefit, with AI applications reducing anxiety through their constant availability and increasing motivation with gamified elements and positive reinforcement. Studies have shown that such support mechanisms are effective in maintaining a positive emotional state conducive to learning [63]. In addition, collaborative creativity facilitated by AI applications supports group projects and peer interactions, fostering a sense of community and collective problem-solving. This collaborative environment aligns with findings from Graesser et al. [64], who emphasized the role of technology in enhancing collaborative learning and creativity.

The provision of accessible learning resources by AI applications supports continuous learning and inspires creativity by allowing students to explore diverse materials anytime. This accessibility is crucial for fostering an inclusive learning environment, as highlighted by Yenduri et al. [65], who noted that diverse and readily available resources enhance educational equity and creativity. Finally, enhanced academic emotions resulting from AI integration create positive learning experiences and build emotional resilience. Adaptive learning paths and supportive environments provided by AI applications contribute to improved emotional well-being and academic performance. This is supported by research indicating that adaptive learning technologies positively impact student emotions and resilience [5–9].

The integration of AI in education has elicited varied responses from both teachers and students, reflecting a complex interplay of benefits and challenges. One prominent concern is the potential for AI applications to impose rigid frameworks that may stifle creativity. This apprehension aligns with the notion that while AI can provide structured guidance, it may also limit the spontaneous and divergent thinking essential for creative processes. This balance between structure and freedom is critical, as noted in the literature on educational methodologies and creativity development [1–3].

Emotional disengagement emerges as another significant issue, with both groups expressing that AI interactions often lack the personal touch necessary for effective learning experiences. The importance of human elements in education is well-documented, with studies emphasizing the role of personal connection in fostering student engagement and motivation [4, 5]. This emotional component is vital, as AI systems, despite their capabilities, may only partially replicate the nuanced and empathetic interactions provided by human educators [6, 7].

Performance anxiety due to frequent AI assessments is another shared concern. AI’s ability to provide continuous and immediate feedback can be a double-edged sword, potentially leading to increased stress and anxiety among students. This is consistent with findings that highlight the psychological impact of constant monitoring and assessment, which can detract from the learning experience and affect student well-being [8, 9].

Technical issues associated with AI applications also pose significant challenges. Both teachers and students have reported frustrations with technical glitches disrupting the learning process. These disruptions can hinder the seamless integration of AI into educational environments, underscoring the need for robust and reliable technology infrastructure [10, 11].

Despite these concerns, both groups recognize the benefits of AI, particularly in enhancing creativity and engagement. AI’s ability to stimulate problem-solving skills and foster creativity is acknowledged as a significant advantage. This aligns with research suggesting that AI can catalyze creative thinking by providing novel applications and approaches to problem-solving [12–14]. Additionally, the literature supports AI’s potential to increase student engagement through interactive and personalized learning experiences [15, 16].

The role of AI in providing personalized feedback is highly valued, with both teachers and students appreciating its capacity to tailor educational experiences to individual needs. Customised learning, facilitated by AI, can address diverse learning styles and paces, thereby enhancing educational outcomes [17, 18]. This personalization is crucial in meeting the unique needs of each student, fostering a more inclusive and effective learning environment [19, 20].

AI’s contribution to collaborative creativity and accessible learning resources is also positively viewed. AI’s ability to facilitate group projects and provide a wide range of learning materials supports collaborative learning and resource accessibility, which are essential components of a modern educational framework [21–23]. Moreover, the enhancement of academic emotions through AI-driven learning experiences highlights AI’s potential to create positive and engaging educational environments [24, 25].

In conclusion, the attitudes of teachers and students towards AI in education reflect a balanced perspective that acknowledges both its limitations and advantages. While there are valid concerns about emotional disengagement, ethical issues, and performance anxiety, the benefits of enhanced creativity, engagement, and personalized feedback cannot be overlooked. This underscores the need for thoughtful and strategic integration of AI in educational settings, ensuring that its deployment maximizes benefits while mitigating potential drawbacks. As AI continues to evolve, ongoing research and dialogue will be essential in navigating its role in education and optimizing its impact on teaching and learning [26–28].

## Conclusions and implications

The integration of AI in educational applications presents a complex landscape characterized by significant challenges and notable benefits impacting students’ creativity and academic emotions. On the downside, AI applications often impose rigid frameworks that constrain creative thinking and innovation, echoing previous research on the stifling effects of structured learning paths. Emotional disengagement is another critical issue, as the repetitive and impersonal nature of AI interactions can diminish student motivation and engagement. This phenomenon underscores the importance of human interaction for maintaining emotional connections in learning. Additionally, the constant monitoring and assessments by AI applications heighten performance anxiety, negatively affecting student well-being. Technical frustrations due to frequent glitches and complex interfaces further disrupt the learning process. At the same time, an over-reliance on AI can reduce critical thinking and self-initiative, hindering essential problem-solving skills. The digital divide exacerbates educational disparities, highlighting the need for equitable access to technology. Ethical concerns about biases in AI algorithms also raise questions about fairness and equity in educational evaluations.

Conversely, AI integration offers substantial benefits, including the stimulation of creativity and enhanced engagement. AI applications can introduce new ideas and improve problem-solving skills, fostering innovative learning approaches. Their interactive nature makes learning more enjoyable and motivating, significantly boosting student participation. Personalized feedback from AI applications offers tailored guidance and immediate responses, helping students improve their work and build confidence. AI applications also provide emotional support, reducing anxiety through constant availability and enhancing motivation with gamified elements and positive reinforcement. They facilitate collaborative creativity, fostering a sense of community and collective problem-solving. Additionally, AI applications offer accessible learning resources, supporting continuous learning and inspiring creativity, which is crucial for educational equity. Adaptive learning paths and supportive environments provided by AI applications improve emotional well-being and academic performance, fostering positive learning experiences and building emotional resilience. Balancing these benefits with the challenges requires thoughtful implementation and continuous evaluation to optimize AI’s role in education.

### Limitations and suggestions for further studies

Despite the merits and rich data, this study has some limitations which need to be mentioned. Firstly, the exclusive use of interviews for data collection limits the breadth of perspectives gathered. Interviews may reflect individual viewpoints rather than broader trends or consensus among participants. Additionally, the absence of focus groups in data collection further restricts the depth of insights obtained, as group dynamics and interactions that could reveal shared experiences or divergent opinions are not explored. Moreover, the study lacks detailed demographic information about participants, such as their majors, teaching experience (for teachers), or other relevant characteristics. This omission must include a nuanced understanding of how these factors might influence perceptions of AI-integrated educational applications.

Furthermore, the study’s small sample size raises concerns about the generalizability of findings. With a limited number of participants, the variability in perceptions and attitudes towards AI in education may need to be adequately captured. Additionally, a comparative analysis between teachers’ and students’ perceptions and attitudes needs to be conducted to uncover potential differences or similarities that could provide richer insights into the impact of AI on educational experiences from both perspectives.

Suggestions for future research include employing mixed-methods approaches that combine interviews with other qualitative methods, such as focus groups. This would allow for a more comprehensive exploration of diverse perspectives and enable researchers to triangulate findings for greater validity. Moreover, expanding the sample size and ensuring diversity among participants in terms of academic disciplines, teaching experience, and student backgrounds could provide a more robust basis for generalizing findings. Additionally, conducting comparative analyses between different stakeholder groups (e.g., teachers vs. students) would deepen understanding of how AI-integrated educational applications affect various participants differently. Finally, longitudinal studies could track changes in perceptions and attitudes over time as AI technologies in education continue to evolve, offering insights into the long-term impacts and adaptations within educational settings. These methodological enhancements and research directions would contribute to a more comprehensive understanding of the complex interactions between AI technology and educational practices.

## Electronic supplementary material

Below is the link to the electronic supplementary material.

Supplementary Material 1

## Data Availability

Data is provided within the manuscript or supplementary information files.
