# Supplementary material for: Artificial intelligence (AI) -integrated educational applications and college students’ creativity and academic emotions: students and teachers’ perceptions and attitudes
Source: BMC Psychol. 2024 Sep 16;12:487. doi: 10.1186/s40359-024-01979-0 (PMC11403842; doi:10.1186/s40359-024-01979-0)
Supplement: Supplementary file 1 — Supplementary Material 1 [file 40359_2024_1979_MOESM1_ESM.docx]

For Students:

How do you perceive AI-integrated educational applications in fostering your creativity compared to traditional methods?

Could you share any specific instances where AI-driven tools have positively influenced your creative process in learning?

Have you experienced any challenges or limitations while using AI-integrated educational applications in nurturing your creativity? If so, what are they?

How do you think AI-powered tools affect your emotional engagement with academic tasks? Do they enhance or detract from your motivation and emotional well-being?

In what ways do you believe AI technology could be further developed to better support your creative expression and emotional experiences in learning?

Can you describe any differences in your learning experience when using AI-based educational tools versus conventional methods in terms of fostering positive academic emotions?

For Teachers:

From your perspective, what are the primary benefits of incorporating AI-integrated educational applications into college curricula in terms of fostering students' creativity?

Have you noticed any changes in students' academic emotions since the implementation of AI-driven educational tools? If so, what are they, and how do they compare to traditional teaching methods?

In what ways do you think AI technology can be leveraged to address challenges related to students' emotional engagement with academic material?

How do you balance the integration of AI-powered tools with more traditional teaching methods to ensure optimal student engagement and emotional well-being?

Can you share any examples of innovative approaches or best practices in utilizing AI-driven educational applications to enhance students' creative thinking and emotional experiences in your courses?

What are your thoughts on potential ethical considerations or concerns regarding the use of AI in educational settings, particularly concerning students' creativity and emotional development?

AI-related Challenges

1. Creativity Constraints: "AI tools limit my creative thinking in educational applications."
   - 1 = Strongly Disagree
   - 2 = Disagree
   - 3 = Neutral
   - 4 = Agree
   - 5 = Strongly Agree
2. Emotional Disengagement: "I feel emotionally disengaged when using AI in educational applications."
   - 1 = Strongly Disagree
   - 2 = Disagree
   - 3 = Neutral
   - 4 = Agree
   - 5 = Strongly Agree
3. Performance Anxiety: "I experience anxiety about relying on AI for academic performance."
   - 1 = Strongly Disagree
   - 2 = Disagree
   - 3 = Neutral
   - 4 = Agree
   - 5 = Strongly Agree
4. Technical Frustration: "Technical issues with AI tools often hinder my learning process."
   - 1 = Strongly Disagree
   - 2 = Disagree
   - 3 = Neutral
   - 4 = Agree
   - 5 = Strongly Agree
5. Over-reliance on AI: "I rely too much on AI for completing my educational tasks."
   - 1 = Strongly Disagree
   - 2 = Disagree
   - 3 = Neutral
   - 4 = Agree
   - 5 = Strongly Agree
6. Digital Divide: "The lack of access to AI technology limits my educational opportunities."
   - 1 = Strongly Disagree
   - 2 = Disagree
   - 3 = Neutral
   - 4 = Agree
   - 5 = Strongly Agree
7. Ethical Concerns: "I have ethical concerns about using AI in my educational activities."
   - 1 = Strongly Disagree
   - 2 = Disagree
   - 3 = Neutral
   - 4 = Agree
   - 5 = Strongly Agree

Factor 2: AI-related Benefits

1. Stimulated Creativity: "AI tools stimulate my creative thinking in educational tasks."
   - 1 = Strongly Disagree
   - 2 = Disagree
   - 3 = Neutral
   - 4 = Agree
   - 5 = Strongly Agree
2. Increased Engagement: "AI applications increase my engagement in academic activities."
   - 1 = Strongly Disagree
   - 2 = Disagree
   - 3 = Neutral
   - 4 = Agree
   - 5 = Strongly Agree
3. Personalized Feedback: "AI provides personalized feedback that enhances my learning."
   - 1 = Strongly Disagree
   - 2 = Disagree
   - 3 = Neutral
   - 4 = Agree
   - 5 = Strongly Agree
4. Emotional Support: "AI tools offer emotional support that helps me in my studies."
   - 1 = Strongly Disagree
   - 2 = Disagree
   - 3 = Neutral
   - 4 = Agree
   - 5 = Strongly Agree
5. Collaborative Creativity: "AI applications enhance my ability to work collaboratively on creative projects."
   - 1 = Strongly Disagree
   - 2 = Disagree
   - 3 = Neutral
   - 4 = Agree
   - 5 = Strongly Agree
6. Accessible Learning Resources: "AI makes a variety of learning resources more accessible to me."
   - 1 = Strongly Disagree
   - 2 = Disagree
   - 3 = Neutral
   - 4 = Agree
   - 5 = Strongly Agree
7. Enhanced Academic Emotions: "AI-integrated educational applications positively influence my academic emotions."
   - 1 = Strongly Disagree
   - 2 = Disagree
   - 3 = Neutral
   - 4 = Agree
   - 5 = Strongly Agree

**Table 1: Factor Loadings After Reduction to Two Factors**

| Item | Factor 1: Constraints | Factor 2: Merits |
| --- | --- | --- |
| Creativity Constraints | 0.85 |  |
| Emotional Disengagement | 0.88 |  |
| Performance Anxiety | 0.82 |  |
| Technical Frustration | 0.79 |  |
| Over-reliance on AI | 0.73 |  |
| Digital Divide | 0.76 |  |
| Ethical Concerns | 0.70 |  |
| Stimulated Creativity |  | 0.82 |
| Increased Engagement |  | 0.85 |
| Personalized Feedback |  | 0.80 |
| Emotional Support |  | 0.79 |
| Collaborative Creativity |  | 0.76 |
| Accessible Learning Resources |  | 0.78 |
| Enhanced Academic Emotions |  | 0.83 |

**Table 2: Reliability Statistics**

| Factor | Number of Items | Cronbach's Alpha |
| --- | --- | --- |
| Constraints | 7 | 0.91 |
| Merits | 7 | 0.89 |

**Interpretation**

- **Factor Loadings:** The loadings show that each item predominantly loads onto one of the two factors, indicating a clear separation between "Creativity Constraints" and "Enhanced Academic Emotions".
- **Reliability:** Both factors demonstrate high internal consistency, with Cronbach's Alpha values above 0.85, indicating good reliability for both factors.
